# Expression of the anti-Mullerian hormone, kisspeptin 1, and kisspeptin 1 receptor in polycystic ovary syndrome and controlled ovarian stimulation rat models

**DOI:** 10.17305/bjbms.2019.4281

**Published:** 2020-02

**Authors:** Ali Risvanli, Halis Ocal, Necati Timurkaan, Pinar Ipek, Ibrahim Seker, Burak Karabulut

**Affiliations:** 1 Department of Obstetrics and Gynecology, Faculty of Veterinary Medicine, University of Firat, Elazig, Turkey; 2 Department of Pathology, Faculty of Veterinary Medicine, University of Firat, Elazig, Turkey; 3 Yesilhisar Health Vocational School, Univerity of Erciyes, Kayseri, Turkey; 4 Department of Zootechny, Faculty of Veterinary Medicine, University of Firat, Elazig, Turkey

**Keywords:** Anti-Mullerian hormone, kisspeptin, ovarian stimulation, polycystic ovary syndrome, COS, PCOS, KISS-1, KISS1r, GPR54 receptor

## Abstract

Polycystic ovary syndrome represents a significant cause of female infertility. The aim of this study was to investigate the expression of anti-Mullerian hormone (AMH), kisspeptin 1 (KISS-1), and kisspeptin 1 receptor (KISS1r) in rat models of polycystic ovary syndrome (PCOS) and controlled ovarian stimulation (COS). For this purpose, 28 rats were assigned into four groups. Estrus and Diestrus groups consisted of rats in estrus and diestrus phases, respectively, while COS and PCOS groups consisted of rats with induced COS and PCOS, respectively. The serum AMH, KISS-1, and estradiol levels, and ovarian KISS1r levels were analyzed by enzyme-linked immunosorbent assay. Furthermore, histopathological analysis of the ovary tissue was done and ovarian KISS-1 expression was determined by immunohistochemical assay. The results revealed that ovarian KISS1r levels were higher in the Estrus (1271.43±51.98 pg/mL) and COS (1191.43±85.67 pg/mL) groups, compared to Diestrus and PCOS groups. The highest level of AMH was found in the Estrus group (16.91±2.12 ng/mL). The results indicate that AMH had no effect on the development of COS and PCOS, while KISS-1 was found to affect the development of COS in rats.

## INTRODUCTION

Anti-Mullerian hormone (AMH) is routinely used in the assessment of ovarian reserves and function, in the diagnosis of cryptorchidism and anorchidism, and in the evaluation of male gonadal functions at any age. AMH may also be useful for the diagnosis of secondary amenorrhea, as well as the discrimination of hypo- and hypergonadotropic hypogonadism. Moreover, it is the only indicator of ovarian reserve unaffected by the feedback mechanism of the hypothalamic-pituitary-gonadal axis, and can be tested in the follicular and luteal phase [1-3].

Kisspeptin (KISS) has been shown to be synthesized in the testis, ovary, pancreas, intestines, central nervous system, and placenta [4]. The KISS1 receptor (KISS1r), also known as the GPR54 receptor, is also present at the level in different tissues, including the ovarian tissues of rats [5] and humans [6]. The KISS1r is widely available in the central nervous system, located on neurons producing gonadotropin-releasing hormone (GnRH) in the hypothalamus.

KISS directly stimulates GnRH release via KISS1r [7]. The central or parenteral administration of this peptide is reported to strongly induce the hypothalamic-pituitary-gonadal axis and elevate gonadotropin levels in the experimental animals [8-9]. In contrast, the administration of KISS to female rats completely stopped the estrus cycle [10], while *KISS-1* gene expression was determined to be modulated by gonadal steroids in the hypothalamus [9].

Polycystic ovary syndrome (PCOS) is among the most common endocrine disorders, affecting 10-25% of women of fertile age [11]. The etiology and pathogenesis of this multi-factorial disease are not clearly understood. The diagnosis of PCOS is based on the presence of at least two of the following criteria: oligoanovulation, clinical or biochemical hyperandrogenism, and the presence of polycystic ovaries on ultrasonography [12]. In recent years, insulin resistance has also been shown to play a role in the development of PCOS. Patients suffering from PCOS have impaired folliculogenesis owing to increased levels of androgens. They may present with irregular menstruation, hirsutism, acne, alopecia, infertility, or recurrent abortions [13].

Ovarian stimulation increases follicular maturation and raises the chances of pregnancy [14]. The controlled ovarian stimulation procedure is aimed at obtaining multiple and qualified oocytes. Ovulation induction with gonadotropins is used in infertility treatment; however, this procedure may result in the development of severe complications. For example, ovarian hyperstimulation syndrome (OHSS) can develop after ovulation induction with exogenous gonadotropins [14, 15]. The mechanism of OHSS is not clear, and this condition is usually irresponsive to therapy.

The treatment of PCOS includes methods that suppress the menstrual cycle. The OHSS arises only in ovulatory cycles and usually 3 to 6 days after human chorionic gonadotropin (hCG) administration [15]. However, cases developing this condition before or long after the hCG administration have also been reported in the literature. A large number of stimulated follicles and harvested oocytes, presence of PCOS, and high serum estradiol level may also contribute to the development of OHSS [14].

Since the etiology and pathophysiology of PCOS are still not clear, our aim was to explore the role and expression of AMH, *KISS-1*, and KISS1r in rats with induced PCOS and COS.

## MATERIALS AND METHODS

### Animals and groups

A total of 28 female Sprague-Dawley rats, age 8-16 weeks, and the weight of 200-250g were included in the study. The rats were purchased and kept in the animal facility of the Firat University Experimental Research Unit, Elazig, Turkey, in groups, in cages with 12 h : 12 h light-dark regimen and food and water *ad libitum*. Prior to the study, research approval was obtained from the Test Animals Local Ethics Committee of Firat University (15.06.2016-2016/12).

The rats were assigned into four groups as follows: Group 1 (i.e., Estrus group) included animals that were in the estrus phase (n=7); Group 2 (i.e., Diestrus group) included animals in the diestrus phase (n=7)*;* Group 3 (i.e., COS group) included animals with induced COS (n=7); Group 4 (i.e., PCOS group) included animals with induced PCOS (n=7). The estrus and diestrus phases were determined by vaginal irrigation. No synchronization protocol was applied to regulate the estrous cycles.

### Controlled ovarian stimulation procedure

COS was induced as previously described by Musal et al. [16]. Briefly, 40 IU of pregnant mare serum gonadotropin (PMSG) (Folligon, MSD- Animal- Health, Canada) was injected intraperitoneally, followed by 48h later intraperitoneal injection of 20 IU hCG (Chorulon, MSD- Animal- Health, Canada).

### Polycystic ovary syndrome induction

PCOS was induced as previously described by Stener-Victorin et al. [17]. Briefly, 4 mg estradiol valerate (β estradiol 17 valerate; SIGMA, USA) in 0.2 ml sesame oil, was injected into 8-week female rats via the intramuscular route.

### Vaginal irrigation

Vaginal irrigation was performed as described by Risvanli et al. [18]. Irrigations were made with sterile distilled water using a rubber pail and pipette. The liquid obtained after irrigation was placed on a slide and analyzed by light microscopy at 400× magnification. The densities of the superficial, parabasal, intermediary cells in the specimens were rated as +, ++, and +++. The rats with a +++ superficial cell density were considered to be in the estrus state.

### Laboratory analysis

The animals were sacrificed under ether anaesthesia, and blood samples of rats in Estrus and Diestrus groups were collected. Further, blood samples from COS and PCOS groups were obtained 2 months after the induction procedure and development of the induced COS and PCOS. The sera of the animals were separated and stored at -20°C until analysis. Left ovaries were used for the analysis of KISS1r levels by enzyme-linked immunosorbent assay (ELISA), while the right ovaries were utilized for histopathological examination and immunohistochemical analyses.

The AMH levels in the blood sera were determined by ELISA, using ELISA reader (BioTek Instruments, ELx800 manual, USA) using a commercial ELISA kit (YL Biont Shanghai YLA0062RA; with a sensitivity of 0.051 ng/ml). Furthermore, KISS-1 levels in the blood sera were measured by an ELISA reader (BioTek Instruments, ELx800 manual, USA) using a commercial ELISA kit (Li StarFish Italy EA0873Ra; with the sensitivity of 2.49 pg/ml). Furthermore, the KISS1r level was analyzed in homogenized left ovaries by an ELISA reader (BioTek Instruments, ELx800 manual, USA) using a commercial ELISA kit (YL Biont Shanghai YLA1199RA; with the sensitivity of 4.98 pg/mL). Briefly, the tissues were stored at -20°C and retrieved to be homogenized in ice molds at 4°C with 0.01 M phosphate buffer saline (pH=7.4) at a 1:10 dilution. Following the homogenization procedure, the supernatants were separated by centrifugation at 3,000 rpm for 10 min, and used in the analyses. Estradiol levels in the blood sera were determined by an ELISA reader (BioTek Instruments, ELx800 manual, USA) using a commercial ELISA kit (Eastbiopharm PRC CK-E10719; with a sensitivity of 1.54 ng/l).

### Histopathological analysis

For the purpose of histopathological analysis, the right ovaries were excised and then fixed in 10% buffered formalin solution. Paraffin blocks were prepared by routine procedures. Five sections (5 µm thickness) were taken from paraffin blocks for each subject, stained with hematoxylin and eosin (H&E), and examined with the light microscope. Total counts of secondary follicles, tertiary follicles, atretic follicles, and corpora lutea were documented on the five slides prepared for each subject. In addition, inflammation, fibrosis, fat infiltration, pigmentation, follicular cysts, and luteinized cysts were recorded in each section.

### Immunohistochemical analysis

To determine the *KISS-1* positive cell distribution in the ovaries, the tissues were immunohistochemically stained using the avidin-biotin complex (ABC) method with a Mouse and Rabbit-specific HRP/AEC detection kit (abcam, Cambridge, UK). Staining was carried out according to the manufacturer’s protocol. A Rabbit Anti-KISS 1/KISS Polyclonal Antibody (bs-0749R, Bioss Antibodies, Boston, USA) was used as the primary antibody. The 5 µ thick tissue sections were placed on poly-L-lysine-coated slides. After the deparaffinization with xylol and rehydration in graded alcohols, the tissues were incubated in a microwave oven for 20 min in a citrate buffer solution for antigen retrieval.

To inactivate endogenous peroxidase, the sections were incubated for 10 min in a 3% hydrogen peroxide solution. The tissues were washed with phosphate-buffered saline (PBS) solution three times in 5-min intervals; subsequently, the tissues were incubated for 10 min in normal serum. Without washing, the tissues were incubated in the primary antibody with a 1/200 dilution at +4ºC overnight. In the next stage, they were washed in PBS and incubated for 10 min in a peroxidase-conjugated biotinylated anti-rabbit solution.

The tissues were again washed three times in PBS and incubated for 30 min with a horseradish peroxidase conjugate for 30 min, followed by treatment with aminoethyl carbazole (AEC) chromogen. Mayer hematoxylin was used for counter-staining. The tissues were scored negative, 1+, 2+, and 3+ when observing no, light, moderate, or intensive staining, respectively. For the negative control, a non-immunized serum was used instead of the primary antibody.

### Statistical analysis

Statistical analyses were performed using SPSS software, version 22.0 [19]. The data were analyzed by descriptive statistics. Kruskal-Wallis test was used to compare the data between the groups. Furthermore, the post-hoc corrected Bonferroni Mann-Whitney U test was used when appropriate. *P* < 0.05 was considered statistically significant.

## RESULTS

The KISS1r levels in the left ovaries were higher in the Estrus group (1271.43±51.98 pg/mL) and COS group (1191.43±85.67 pg/mL) compared to Diestrus and PCOS groups (*p*<0.05).

The highest AMH level was observed in the Estrus group (16.91±2.12 ng/mL) (*p* < 0.05). The results revealed no significant difference between the groups with regard to plasma *KISS-1* and estradiol concentrations (Table 1).

**TABLE 1:**
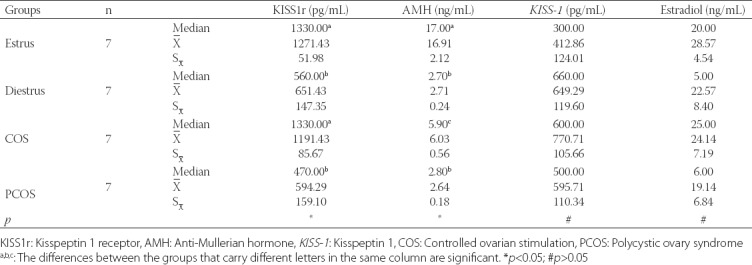
Kisspeptin-1 receptor, anti-Mullerian hormone, kisspeptin-1, and estradiol concentrations in plasma

**FIGURE 1:**
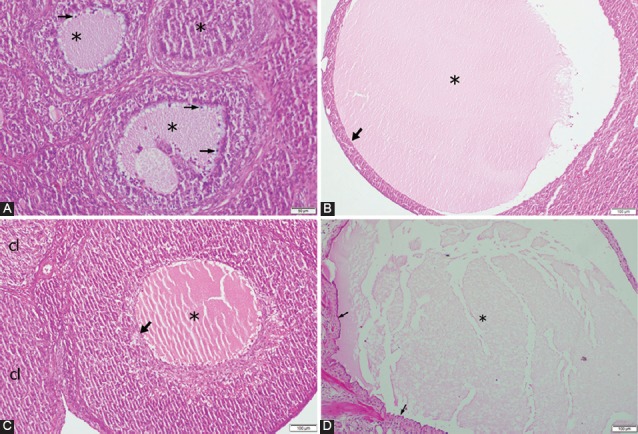
Histopathological changes in the ovaries; A) ovary of a rat in the polycystic ovary syndrome group showing atretic follicles (stars), disorganization of granulosa cells, and picnotic granulosa cell nuclei (arrows), H&E; B) ovary of a rat in the controlled ovarian stimulation (COS) group showing follicle cysts, lumen of the cyst (star), and wall of the cyst (arrow), H&E; C) ovary of a rat in the COS group showing luteinized cysts, lumen of the cyst (star), and wall of the cyst (arrow) along with normal corpora lutei (cl), H&E; D) ovary of a rat in the COS group showing surface inclusion cysts, lumen of the cyst (star), and the epithelial layer (arrows), H&E.

The results of the histopathological examination of the ovaries are summarized in Table 2. The COS group had the lowest number of the tertiary follicles (1.71±0.52; *p*=0.002, while, the PCOS group was found to have the highest number of atretic follicles (5.86±0.91; *p*=0.003; Figure 1A) and the lowest number of corpora lutea (6.00±2.44; *p*=0.049; Table 2). There was no significant difference among the groups regarding the number of secondary follicles. In the COS group, luteinizing cysts were detected in five animals, while follicular cysts and surface inclusion cysts were observed in one animal (Figure 1B-D). With regard to the PCOS group, oophoritis and perioophoritis were observed in one case. However, no abnormal findings were encountered in the ovaries of the animals in the Diestrus and Estrus groups.

**TABLE 2:**
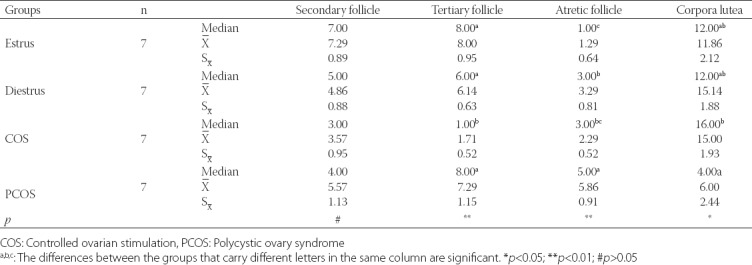
Histological examination findings of the ovaries

**FIGURE 2:**
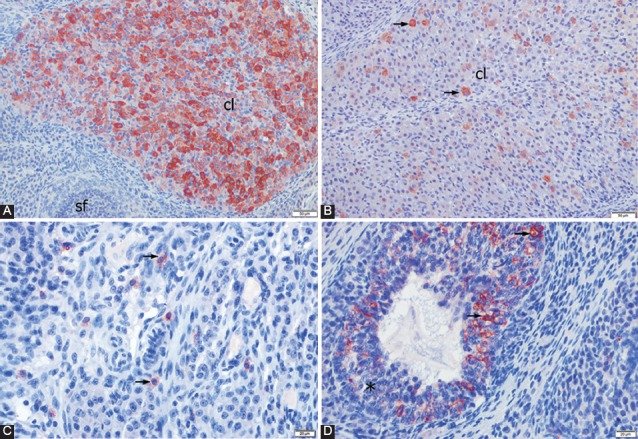
Immunohistochemical localization of kisspeptin in the ovaries; A) ovary of a rat in the controlled ovarian stimulation (COS) group showing intensive positive staining (red) in the luteal cells of the early-stage corpora lutea (cl), ABC method, Mayer’s hematoxylin counterstain, AEC chromogen; B) ovary of a rat in the polycystic ovary syndrome group showing weak positive staining (red) in the luteal cells of the late-stage corpora lutea (cl); ABC method, Mayer’s hematoxylin counterstain, AEC chromogen, C) ovary of a rat in the diestrus group showing immunopositive staining in the cytoplasm of the interstitial cells (arrows). ABC method, Mayer’s hematoxylin counterstain, AEC chromogen; D) ovary of a rat in the Estrus group showing immunopositive staining (arrows) in the granulosa cells of the atretic follicles (star). ABC method, Mayer’s hematoxylin counterstain, AEC chromogen.

The immunohistochemical localization of KISS was determined based on the presence or absence of red cytoplasmic staining. Staining with nonimmune serum was used as a negative control. The most intense staining was observed in the corpora lutea. However, staining was denser in the early-stage corpora lutea (Figure 2A) and less dense in the late-stage corpora lutea (Figure 2B). The immune reactivity in the corpora lutea was at its densest level in the COS and Diestrus groups, followed by the Estrus and PCOS groups.

Positive staining was also encountered in the interstitial cells (Figure 2C), granulosa cells of atretic follicles (Figure 2D), and zona pellucida. Furthermore, there was weakly positive staining in the oocytes. Positive staining was not determined in the granulosa cells of primordial, primary, secondary, and tertiary follicles in all cases of the experimental groups. The immunohistochemical localization and staining density of KISS in the ovaries of each study group are presented in Table 3.

**TABLE 3:**
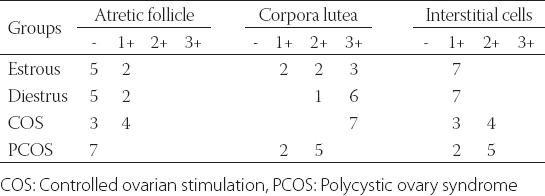
Immunohistochemical localization and staining density of kisspeptin in the ovaries in each study group

## DISCUSSION

In a study investigating plasma AMH levels as a marker of the preantral follicle population [20], KISS was shown to increase the plasma AMH in 6- and 10-month rats. In the same study, KISS administration was proposed to increase the number of small antral follicles, thereby enhancing AMH concentration, which is known to be released from secondary and small antral follicles.

The reason for AMH elevation in human patients with PCOS is not clearly understood. Accordingly, the role of AMH in the pathogenesis of PCOS has not been known yet. The hypomethylation of the *AMH* gene may lead to the intrinsic oversynthesis of the *AMH* gene and increased AMH production in patients with PCOS. Increased AMH concentration leads to anovulation by inhibiting the follicle-stimulating hormone release that stimulates follicle growth [2, 3]. Serum AMH concentration increases depending on the growth in the preantral and small antral follicle count in women with PCOS. This condition is more evident in women with anovulatory PCOS depending on the increase in the number of small antral follicles [21].

In a study performed by Emekci Ozay et al. [22], serum AMH concentration was reported as 5.93±4.93 ng/mL in women with PCOS, which was higher than the value obtained for healthy women. In the same study, the serum KISS concentration was found to be higher in women with PCOS, compared to that in healthy women; however, this difference was not statistically significant. The authors suggested that KISS increased the luteinizing hormone (LH) concentration via GnRH and that the LH concentration was higher in women with PCOS. Regarding this, they concluded that the high KISS concentration in women with PCOS was normal.

Cimino et al. [1] reported that an important subgroup of GnRH neurons expresses the AMH receptor in mice and humans and that AMH may activate GnRH neuron stimulation in mice. They hypothesized that AMH increases GnRH-related LH pulsatility and has a central effect on GnRH neurons. In addition, it was assumed that the AMH-related regulation of the GnRH release may play a role in the pathophysiology of PCOS.

According to animal studies, inappropriate exposure to sex steroids, mainly excessive androgen, could be associated with PCOS pathogenesis in the early stages of development. One mechanism contributing to the neuro-endocrinological changes in PCOS is the interruption in the KISS-1 system, particularly due to steroids [3]. In a number of studies, it was demonstrated that the PCOS-like metabolic disorders and reproductive dysfunctions, as seen in PCOS, arose in the female rats administered with androgenic agents. This condition may be associated with the organizing effect of sex steroids on the *KISS-1* system and proper maturation of *KISS-1* neuronal population, playing a key role in the sexual differentiation of the brain [23, 24].

In a study investigating the PCOS rat model, the KISS-positive cell number was shown to increase in rats with PCOS [25]. However, all papers stress that the pathogenesis of PCOS remains unclear. In the present study, although the mean plasma *KISS-1* level was 595.71±110.34 pg/mL, the difference among the groups was not statistically significant. Although the KISS1r concentration was at the highest level in the Estrus (1271.43±51.98 pg/mL) and COS groups (1191.43±85.67 pg/mL), it was determined as 594.29±159.10 pg/mL in the PCOS group. In addition, the AMH concentration was at its maximum level in the estrus group (16.91±2.12 ng/mL). Our findings regarding the lower levels of KISS1r and AMH in the PCOS group, compared with the results reported by other researchers, may not explain the pathogenesis of PCOS. This discrepancy may be due to the difference in the species studied.

Ovarian hyperstimulation syndrome, which particularly develops after in vitro fertilization (IVF) procedures, is among the most severe complications emerging after ovarian stimulation and may be life-threatening. However, the pathogenesis of this syndrome has not been clearly understood yet [26]. The OHSS was reported to be associated with high serum AMH levels before COS [27]. La Marca et al. [28] reported that all patients whose IVF cycle was canceled due to poor response to controlled ovarian stimulation had a low serum AMH level. Nonetheless, patients whose IVF cycle was stopped due to OHSS risk had the highest serum AMH levels. These data suggest that AMH may be useful for the prediction of OHSS for IVF cycles besides being a proper marker for assessing the ovarian reserves [26].

In the present study, the mean plasma *KISS-1* level was determined as 770.71±105.66 pg/mL in the COS group and denser immune reactivity was seen in the corpora lutea; however, the difference was not significant. Zhai *et al*. [29] reported that KISS inhibits OHSS by suppressing vascular endothelial growth factor secretion. The highest level of KISS1r concentration in the ovaries were found in the Estrus (1271.43±51.98 pg/mL) and COS (1191.43±85.67 pg/mL) groups. Although the AMH concentration was at its maximum in the estrous group (16.91±2.12 ng/mL), it was higher in the COS group (6.03±0.56 ng/mL) than in the PCOS group (2.64±0.18 ng/mL).

In conclusion, there was no evidence regarding the presence of a relationship between AMH level and PCOS and/or COS in rats. However, an elevation was observed in KISS and KISS1r levels in rats with COS suggesting that the involvement of KISS in follicular development in the ovaries. Nonetheless, further studies are required to investigate this issue in other animal species.
